# Individual Stress Prevention through Qigong

**DOI:** 10.3390/ijerph17197342

**Published:** 2020-10-08

**Authors:** Karen van Dam

**Affiliations:** Faculty of Psychology, Open University of the Netherlands, 6419 AT Heerlen, The Netherlands; karen.vandam@ou.nl

**Keywords:** stress prevention, recovery, well-being, Qigong, meditative movement

## Abstract

Owing to work intensification and an accelerated pace of life in general, individuals in many Western countries are often overactivated and find it difficult to switch off. However, recovery from physiological and mental activation is critical to prevent stress symptoms and maintain one’s physiological and mental well-being. Extensive research evidence indicates that Qigong, a traditional Chinese movement practice for promoting health, provides an effective means to recover from work and off-work demands. The main objective of this paper is to offer a comprehensive, narrative review of the effects of Qigong and its core components. Attention is first paid to the outcomes of work and off-work demands and stress, and the role of recovery for individuals’ well-being. Then, Qigong and its components are explained, followed by the results of scientific research. Finally, limitations and implications for research and practiced are discussed.

## 1. Introduction

Over the past decades, stress has become an urgent health issue affecting individuals, organizations, and society. With globalization driving ever-changing and fast-paced working conditions, the speed of working life has increased over the years, leaving employees less time for more tasks with higher cognitive and emotional demands [1,2]. This intensification of working life is part of an accelerating culture and a general experience of day-to-day life as being rushed and hurried [3]. Owing to technology, such as mobile phones and the internet, work has invaded the private sphere as employees are always available [4]. In addition, social media has become an important means for information and communication, requiring constant attention and immediate responses [5]. As a result, we are always busy and activated, even in our leisure time.

This activation comes at some price, however. Research has provided strong evidence for the adverse effects of chronic stressors and demands on individuals’ physical and psychological health [6,7,8]. Chronic overactivation can instigate a negative spiral in which an overloaded mind and body can diminish individuals’ ability to “switch-off” at night, thus lowering sleep quality and increasing exhaustion [9]. Moreover, stress sensitization can develop, causing overactivated individuals to overreact to stressful situations [10] and even create new stress situations, as stressed people might be less able to deal with situations adequately [11].

Recovery from physiological and mental activation is important for the physiological system to unwind and return to its setpoints [7,12] and for the mind to come to rest [13]. Findings show that both off-job recovery experiences [14,15] and within-work breaks [16] relate to improved physical and mental well-being. Despite increasing evidence that unwinding is important for individuals’ well-being and performance [17], interventions aimed at enhancing unwinding and recovery are still scarce [15]. Given the aging population and rising retirement ages, workers will have to take preventive actions themselves to stay healthy during their extended career. An important question therefore arises: what can workers do to relax from work and daily activation, restore the balance in body and mind, and thus prevent strain, ill-health, and drop-out?

Research indicates that Qigong is an effective means of recovering from chronic work and non-work requirements and thus can serve as an important, yet simple way to prevent health problems from occurring [18]. Qigong is a traditional Chinese movement practice for promoting health that includes slow movements, breathing exercises and meditation [19]. Systematic research with randomized control trials (RCTs) provides evidence of the positive effects of Qigong on a range of health indicators, such as high blood pressure, sleep quality, and general well-being (e.g., [18,20,21]).

The goal of this paper is to provide a comprehensive, narrative review of the effects of Qigong and its elements for health and well-being. To better understand the impact of Qigong, attention is first paid to the impact of work and off-work demands and stress, and the role of recovery for well-being. After explaining Qigong and its components, the results of scientific research investigating their impact is presented. Finally, limitations and implications for research and practice are discussed.

## 2. Stress, Sustained Activation, and Recovery

### 2.1. Stress and Sustained Activation

Both stressful and continuous demands can instigate a physiological response that may undermine individuals’ health and well-being. Stress was originally viewed as a response of the body to an objective, aversive stimulus, such as electric shocks, hunger, and heat, with the goal of regaining homeostasis [22]. The two world wars made researchers aware of the role of subjective, psychological stressors [23]. According to appraisal theories [24,25], stress situations instigate an appraisal process in which people evaluate the possible impact of a situation and whether they are able to cope with it. A stress response occurs when people think they have insufficient options to deal with a threatening stressor. Although appraisal models have been criticized for their emphasis on cognitive processes, it is generally acknowledged that objective stressors exert their effects primarily through individuals’ subjective perceptions and interpretations [23].

In the field of occupational health psychology, researchers have established various work characteristics that relate to stress and well-being [26,27]; especially, characteristics that hinder the obtainment of personal work goals impact well-being [28]. As work characteristics vary little over time, most stress situations at work are chronic. Day after day, employees experience, for instance, high work load, time pressure, a negative working climate, continuous interruptions and other irritations; events that are not life-threatening in themselves but do lead to chronic activation of the stress system, and it is often the long-term effects that can have adverse consequences [7,29]. Employees may also respond to anticipated or imagined stressful events that have not (yet) occurred, or worry about the work situation, implying that the stress situation continues when they are at home [9].

In addition to perceived and anticipated work stress, there are many events and activities, on and off the job, that require physical and psychological activation without being a typical stressful situation. Meeting those demands is always taxing and requires effort, even when these demands are enjoyable and challenging [28]. As the day progresses, the energetic state available to perform these tasks decreases, and compensatory effort is needed to maintain required performance levels [12]. Although exposure to such tasks may not necessarily elicit a stress and appraisal process, it does require physiological and psychological activation. In fact, challenging and enjoyable job demands have been related to increased problem-solving pondering during nonwork time [30], and less relaxation and detachment from work [14]. In addition, the introduction of the internet and social media has had an impact on how people spend their leisure time [5]. While the internet and social media have become important means for information and communication, the extensive use of these media can pose extra demands on the users’ physiological system and hinder the recovery process. A substantial body of evidence has linked computer and internet use in general, and social media use specifically, to poor sleep quality [31,32]. Also, heavy smartphone use has been associated with anxiety and depression [33]. Given this situation, it is not surprising when people are overactive and find it difficult to relax. However, if this situation persists, health damage can eventually occur [7].

### 2.2. Physiological Responses to Stress

What happens when individuals are confronted with stressors or extended periods of activation? To meet the demands on and off work, the body reacts with the activation of a fast sympathetic system and a hormonal system, the hypothalamic-pituitary-adrenal (HPA) axis [34,35,36]. In the first, rapid response, the hypothalamus sends a signal to the adrenal glands triggering the release of catecholamines, including the hormones adrenaline and noradrenaline. These hormones immediately activate the sympathetic nervous system, causing (among others) the heart rate to increase in frequency and contraction force, the blood to transport more oxygen, glucose, and fatty acids, increased muscle tension, and suppression of parasympathetic system activities, such as the immune system. At the same time, a hormonal system, the HPA axis, is activated in which the hypothalamus stimulates the pituitary gland, through the corticotrope-releasing hormone (CRH) to produce the adrenocorticotropic hormone (ACTH), which then triggers the adrenal cortex to secrete cortisol [34]. Cortisol supports the activities of the catecholamines by contributing to energy mobilization through increased glucose availability and by suppressing the highly demanding metabolic processes of the immune system, resulting in further availability of glucose [35]. This second, HPA response develops a little slower than the first, neural response but then becomes the dominant response, especially in chronic stress and activation situations. The HPA axis plays a central role in regulating a large number of homeostatic systems in the body, including the metabolic system, cardiovascular system, immune system, reproductive system, and central nervous system [36].

These sympathetic and HPA-axis responses are adaptive reactions of the body, enabling the individual to adjust effectively to the environment, after which the responses slowly disappear and homeostasis is restored [7]. However, the constant need to deal with stressors and other demands can lead to a cumulative physiological burden, or allostatic load [37], that can cause structural changes in biological systems and eventually result in diseases [29,34,38]. The chronic production of cortisol, for instance, is associated with a number of health problems, such as reduced functioning of the immune system [39], and increased risk of diabetes [35]. Moreover, excessive cortisol secretion can cause disruption in the feedback inhibition loop, whereby cortisol inhibits its own production through reduced production of CRH. In this way, a cascade of hippocampal damage can occur (the “glucocorticoid cascade hypothesis” [38], resulting in further excessive cortisol production [36,40]. More generally, chronic stress and allostatic load are associated with chronically elevated blood pressure (hypertension), increased risk of cardiovascular disease, reduced cognitive functioning and burnout, medical problems such as arthritis and diabetes, gastrointestinal problems, sleep disturbance, and mental disorders such as depression [8,34,37,41]. In particular, the overproduction of ACTH and cortisol can enhance depressive symptoms [42]. These health problems indicate that it is of utmost importance to deactivate the stress system, reduce the stress response, and restore homeostasis.

### 2.3. Homeostasis and Recovery

Homeostasis refers to an optimal, yet dynamic state of internal conditions where all functions in the body (such as body temperature, blood pressure, breathing, muscle tension) are within certain basic limits (homeostatic range). Homeostasis can be achieved by recovering from activation [42]. Recovery refers to “unwinding and restoration processes during which a person’s strain level that has increased as a reaction to a stressor or any other demand returns to its prestressor level” (p. 366, [15]). In the past two decades, researchers have investigated how people can recover from work, with an initial focus on recovery experiences during leisure time [43]. Findings indicate that psychological detachment from work, in particular, is an effective recovery strategy during nonwork time [14,15]. However, immediate effects of leisure time recovery are often short-lived; the recovery process is enhanced when workers are able to let go of work-related thoughts and refrain from job-related activities, although these are often short-lived [44]. Researchers have also found positive effects of recovery options during the working day, such as a lunch break or a “mini-break” whereby attention is briefly removed from work [16]. Especially, activities that require little effort, such as a nap, daydreaming, relaxing, and stretching, appear to have a positive effect [15].

Together, theory and evidence indicate that unwinding from physiological activity is necessary to recover and stay healthy. Research findings additionally suggest that the beneficial effects of longer leisure periods, such as weekends and holidays, often do not last very long, and that shorter breaks can be used for recovery as well [15]. This means that recovery need not be delayed until the weekend or the holiday, and that daily recovery moments can be applied to preserve ones’ health. As such, Qigong could be used as a means of recovery, de-activation, and a return to homeostasis.

## 3. The Role of Qigong for Recovery and Health

### 3.1. What is Qigong?

Qigong is a collective term for a long-established, extensive set of exercises first created in China more than 2000 years ago [18,19]. Qigong is part of traditional Chinese Medicine, a holistic health promotion system that also includes other therapies such as acupuncture, herbs, massage, and nutrition [45]. Central to Qigong is the term “Qi”, the Chinese word for the life energy that is believed to flow through the body. “Gong” means work or benefits acquired through perseverance and practice. Qigong can thus be described as working with the life energy in order to improve one’s health and mind–body balance [19] and is considered a typical preventive means for maintaining good health. While Qigong is an important part of Chinese history and health practices, its appeal and scope extend beyond the confines of traditional Chinese culture [21]. Nowadays, Qigong is practiced around the world as a means for improving health and the mind–body balance. Four components of Qigong exercises in particular appear essential for individuals’ recovery from physiological and psychological overactivation and improved well-being: physical training; active relaxation; focused attention; and conscious breathing [18,46,47,48].

Physical training. Qigong entails performing slow, controlled, soft, and sometimes complex movements at a moderate aerobic level, although some styles also use fast and strong movements. Most movements, however, are smooth and coordinated, with rotations starting from the stomach, and postural changes and weight shifts that seamlessly follow each other [19,49]. Together, the Qigong movements contribute to relaxation, coordination, balance, strength, flexibility, and health [50,51,52]. The saying goes that, through Qigong practice, one will become strong as a woodcutter, flexible as a child, and calm as a sage.

Active relaxation. The Qigong movements must be performed as relaxed as possible, although there are some exceptions. Of course, muscles are tightened, but then they are released again. By actively relaxing the muscles and allowing the full body weight to sink into the earth, unnecessary tension is released, not just physically but also mentally. As Jacobson (p. 3, [53]) claimed: “An anxious mind cannot exist in a relaxed body”. This aspect of Qigong has similarities to muscle relaxation techniques, which are used therapeutically to reduce pain and stress complaints [54].

Focused attention. During Qigong, practitioners’ attention is focused on the movements, body positions, and breathing pattern. This focused attention creates an awareness-in-the-moment (mindfulness) in which past and future are disregarded. For this reason, Qigong is also referred to as “meditation in motion” [19,48,49] and “meditative movement” [18,46]. An important outcome of Qigong is therefore a state of mental peace, or “*Rujing*” [55].

Controlled breathing. Characteristic of Qigong is a conscious and generally calm breathing pattern. The smooth movements in Qigong are synchronized with this calm breathing, for instance, inhaling while slowly raising the arms and exhaling when the arms are slowly lowering [47]. This component of Qigong is related to medical breathing interventions aimed at improving health complaints such as high blood pressure and anxiety [56,57,58,59].

While each component may evoke health benefits, the combination of all four elements may bring more benefits than a single element [47]. During the training, often a reinforcing process takes place, whereby breathing slowly and moving quietly creates increased relaxation, which in turn deepens and slows down breathing and movements even more.

### 3.2. Health Effects of Qigong

In recent decades, scientific research into Qigong has increased as researchers have become increasingly aware of Qigong as a possible intervention for different ailments, such as hypertension, anxiety, and depression (e.g., [59,60,61,62]). Most studies reported below are Randomized Controlled Trials, whereby participants are randomly assigned to either the Qigong intervention group or a control group. Findings indicate that practicing Qigong can provide benefits to stressed and overactivated workers, improving their physical and psychological well-being [18,63].

Hypertension. A consistent outcome of Qigong is lowered blood pressure [51,62,64]. Meta-analytic review studies (e.g., [18,64]) show that practicing Qigong significantly lowers systolic and diastolic blood pressure. For instance, Lee et al. [65] found that a 10-week intervention, in which participants practiced a half-hour Qigong series three times a week, significantly lowered the blood pressure of the Qigong group, while the blood pressure of the control group showed no difference.

Cardiovascular and respiratory systems. Relatedly, Qigong has beneficial effects on the cardiovascular system (heart, blood vessels, and blood) [18]. Increased heart rate variability was found after a 12-week program of one-hour Qigong training three times a week, suggesting increased parasympathetic activity [66]. Practicing Qigong also appears to support the respiratory system as it increases lung capacity, oxygen intake and improves breathing patterns [51,63].

Immune function. Research shows that Qigong has a positive effect on the immune function [67,68] and thus helps reduce the deleterious influence of cortisol. Practicing Qigong is associated with increased numbers and functional activities of white blood cells, including lymphocytes, monocytes, and natural killer cells [68]. Manzaneque et al. [67] observed improved immune functions after a month of a half-hour daily Qigong training program. Yang and colleagues [69] noticed that practicing Qigong enhanced the effect of the flu shot, which also points to improved immune functions.

Stress levels. Practicing Qigong is associated with a reduction in both perceived stress [70,71,72,73] and hormone levels (adrenaline, noradrenaline and cortisol) [65,70]. Studying a group of computer operators, Skoglund and Jansson [74] found that Qigong reduced noradrenaline excretion in urine and influenced the heart rate and temperature, indicating decreased activity of the sympathetic nervous system. Together, these findings suggest that Qigong has an inhibitory effect on the stress response.

Sleep quality and fatigue. Qigong leads to improved sleep quality [63] and a reduction in fatigue and exhaustion complaints [75,76], which are important indicators of burnout [27]. At the end of a nine-week intervention program with two one-hour Qigong training sessions a week, participants reported improved sleep quality and sleep latency (i.e., the time to fall asleep) and less fatigue than before the intervention; this effect was still noticeable after three months [20].

Anxiety. Meta-analytic review studies suggest beneficial effects of Qigong on both momentary (i.e., state) anxiety and chronic (i.e., trait) anxiety [63,73,77]. Johansson and colleagues [77] observed an immediate effect of a single Qigong training on state anxiety. Chow et al. [70] found a long-term effect of an eight-week Qigong training program on participants’ anxiety levels.

Depression. Findings indicate that Qigong can alleviate depressive complaints [20,63,78,79]. Chan et al. [20] reported a reduction in depressive symptoms immediately and three months after a 16-week Qigong intervention. A 10-week Qigong training for nursery and midwifery students resulted in reduced depressive mood [80]. The effectiveness of Qigong in relieving depression has been attributed to a reduction in cortisol levels [42] and activation of the parasympathetic nervous system [81].

Cognitive functioning. Because Qigong is associated with focused attention and a reduced stress response, it may also provide cognitive benefits. A study with healthy, older adults showed that the practice of Qigong was related to an improvement in cognitive functioning that was maintained over a 12-month period [82]. Ladawan and colleagues [47] similarly found that Qigong exercise improved cognitive functioning (attention, brain processing speed, and maximum workload) of healthy practitioners. However, this effect disappeared 12 weeks after discontinuation of Qigong, which suggests that performing Qigong regularly is an important condition for maintaining an effect.

Desensitization and positive appraisal. In addition to diminished physical and psychological responses to stress and sustained activation, it is likely that the condition of mental peace (*Rujing*), achieved through Qigong practice, will help people deal with demanding situations differently. An accumulation of chronic strain and an inability to deactivate sufficiently can result in an increased level of sensitivity to stressors, i.e., stress sensitization [10]; as a result, people may react too strongly to situations and conflicts. As Qigong lowers practitioners’ stress and activation levels, people might be better able to distance themselves from a situation, appraise it more positively [83], and generally show improved emotion regulation [84]. Following, they will experience less mental stress [9], and their responses to situations will be less intense [85]. In this way, Qigong practice may contribute to a positive spiral that enhances individuals’ well-being.

### 3.3. Health Effects of Qigong’s Components

In addition to research that focuses directly on the effects of Qigong, several literature works have studied the effects of the components of Qigong. As this literature may provide more insight into how Qigong works, it is included in this review.

Physical training. There is increasing evidence for the positive effects of physical activities on health and functioning (e.g., [43]). Whereas initially the focus was on heavier physical (aerobic) training, there is now support for the beneficial effects of more moderate physical activities, as is the case with Qigong. Physical activity causes a more active hippocampus through, among other things, an increase in neurotransmitters, improved blood flow, and the production of new nerve cells and longer and more complex dendrites [86]. A more active hippocampus, in turn, has been shown to decrease the harmful influence of cortisol on its’ feedback loop [38], such that less cortisol is produced and the stress response diminishes [40]. As such, physical exercise helps reduce the dysfunctional mechanism as described by the glucocorticoid cascade hypothesis [38]. Moderate physical activity also has a positive effect on the cardiovascular system [87], decreasing sympathetic overactivity and blood pressure [88]. Exercise has a positive influence on cognitive performance as well; especially, the executive functions of the brain that relate to attention, impulse regulation, and working memory benefit from physical activity [89]. To achieve these positive effects, the physical training does not have to be intensive; the movements must be dynamic (using the large muscles for at least 20 minutes), take place at least three times a week, and should be continued, as these positive effects will disappear when people stop exercising [87]. Together, these findings suggest that the physical exercise component of Qigong has benefits for many processes related to stress and overactivation.

Active relaxation. The active relaxation that is emphasized in Qigong resembles other muscle relaxation techniques. Progressive muscle relaxation was introduced by Jacobson [53], who discovered that, by systematically tensioning and releasing different muscle groups and learning to distinguish the resulting sensations of tension and relaxation, people are able to largely eliminate muscle contractions and achieve a sense of deep relaxation [90]. While muscle relaxation techniques are often used for complaints that have a muscle component, such as pain, they also appear effective for reducing physiological activation, anxiety, stress, and heart problems [91,92]. Studying the impact of a 20-minute muscle relaxation program for highly stressed college students, Dolbier and Rush [93] noticed that the muscle relaxation group demonstrated significantly greater increases in mental and physical relaxation, improved heart rate variability, lowered anxiety and cortisol, compared to the control group. Muscle relaxation can also improve sleep quality, with decreased fatigue and increased energy as a result [94]. Together, this evidence suggests that contracting and releasing muscles during Qigong contributes to the well-being of the Qigong practitioner [47,48].

Focused attention. Owing to its focused attention to movements and breathing, Qigong has a direct link with mindfulness and meditation. Mindfulness is a state of mind characterized by deliberate attention to the present moment with an open, non-judgmental awareness of the inner and outer experiences [95]. Mindfulness training includes meditation and other exercises that are aimed at promoting open, non-evaluative perception [96].

Research shows that the effects of mindfulness and meditation are similar to the effects of Qigong, including reduced fatigue, stress, tension, emotional reactivity, rumination, anxiety and depressive symptoms, and improved immunity, sleep quality, and relaxation [96,97,98,99]. In particular, the breath-oriented meditation, more than other forms of meditation, is effective in reducing activation [100]. Meditation also has an influence on a wide scale of cognitive processes, resulting in increased creativity, cognitive flexibility, and problem-solving capacity [101]. More than is the case in Qigong research, mindfulness and meditation studies have focused on the brain [32]. Mindfulness and meditation training appears to cause an increase in alpha and theta brain waves, indicating a relaxed state of alertness that promotes mental health [99], and an increase in left-sided activation in the anterior cortical area, which has a relationship with positive emotions, adaptive responses to stress, and a faster recovery from a stressful provocation [13]. Together, these findings indicate that mindfulness and meditation, and possibly Qigong, can affect functions of the brain, such that deactivation and recovery is enhanced and homeostasis is restored.

Controlled breathing. Most Qigong movements are accompanied by a controlled and slow breathing pattern. During situations of stress and overactivation, breath frequency increases in an effort to quickly distribute oxygen-rich blood to the body [34]. To recover from stress, breathing techniques are used that focus on slowing down the breath frequency [58]. Regular and conscious practice of calm breathing has been shown to promote physical and psychological well-being by reducing sympathetic activation and increasing parasympathetic activation; that is, it decreases stress hormones and hypertension and improves the cardiovascular and respiratory condition [56,57,102,103]. Psychological effects relate to increased comfort, relaxation, vigor and alertness, attention and concentration, and a decrease in arousal, anger, anxiety, and depressive symptoms [56,58,102]. Research also shows that it matters whether one breathes through the nose, as is common practice in Qigong, or through the mouth. When inhaling through the nose, neurons in the limbic system (especially the amygdala, hippocampus, and olfactory cortex) are stimulated; these are brain areas where emotions, memory, and scents are processed. Subjects in Zelano et al.’s [104] study who were breathing through the nose performed better on memory tasks and responded less strongly to emotional stimuli than subjects who were breathing through the mouth.

## 4. Discussion

### 4.1. Summary of Evidence and Comparative Effectiveness

While the adverse effects of stress and sustained activation on health and well-being have been widely established, interventions to reverse and prevent the stress process have received less attention [15]. This narrative research review indicates that Qigong provides an effective approach that individuals can use to recover from stress and sustained activation, regain homeostasis and mind–body balance, and improve their physical and psychological well-being. Table 1 presents a summary of the positive outcomes that have been found in studies investigating the effects of Qigong as a whole and in fields that have addressed important components of Qigong. In order to achieve the desired well-being effects, practitioners should participate in Qigong training on a regular basis and over a longer period of time [20]. These studies achieved significant effects using training schedules with multiple (2–3) sessions per week that lasted between 30 and 60 minutes, over an eight-to-sixteen-week period (e.g., [20,65,66]. The range of outcomes included in this review shows that Qigong provides an effective intervention for reversing the sympathetic and HPA responses that result from stress and sustained activation. Moreover, practicing Qigong may have a buffering effect on health as the state of mental relaxation (or *Rujing*) can help individuals to appraise stressful situations more positively [83], inhibit the development of stress sensitization [10], and take a more relaxed stance in our 24/7 society [19].

Whereas practicing Qigong thus appears to enhance individuals’ physical and psychological health, the question arises whether Qigong is more effective than other interventions? As this review shows, positive effects can also be obtained by practicing one of Qigongs’ components, such as physical exercise and meditation. In addition, other approaches might have positive health effects. Yoga has similarly been found to be effective for reducing stress and health factors associated with high stress, such as hypertension and anxiety [105]. For instance, Maddux et al. [106] noticed how a 16-week yoga program, with two one-hour classes a week, significantly improved university employees’ self-reported stress, depression, insomnia, and general psychological health. As with Qigong, these beneficial effects have been attributed to the role of focused attention and controlled breathing, which help calm the mind while activating the parasympathetic system [102]. Unfortunately, there are only very few comparative studies that include Qigong. In a qualitative, phenomenological study, Raingruber and Robinson [107] found that Qigong, yoga, Reiki, and meditation classes made practitioners, i.e., nurses, aware of sensations of warmth, pulsation, and calm, enhanced problem-solving ability, and increased the ability to focus on patient needs. Comparing Qigong with moderate physical exercise (including walking and callanetics), Taylor-Pillae and colleagues [82] found that the physical exercise group had greater improvements in upper body flexibility, while the exercise group showed greater improvements in balance and cognitive-functioning. Audette et al. [66] compared the effects of two three-month programs for elderly women: a short Tai Chi training versus brisk walking, on several fitness and psychological well-being measures. The findings showed that both groups outperformed a sedentary control group on most indicators. Additionally, Qigong was found to be significantly better than brisk walking in enhancing certain measures of fitness including lower extremity strength, balance, and flexibility. However, Kjos and Etnier [78] observed similar effects of a two-day training of 22 minutes in either medical Qigong or self-paced brisk walking on blood pressure, pulse rate, and affect compared. Also, a short Qigong intervention was found to be less effective in long-term weight loss maintenance compared to an acupressure intervention [108]. It is difficult to draw conclusions from so little evidence; more research is needed to gain more insight into the relative effectiveness and mechanisms of different interventions.

### 4.2. Limitations and Future Research Directions

When interpreting the findings presented in this review, a number of limitations should be taken into consideration. First, due to the limited space available, this narrative review could only report on a selection of individual and meta-analytical evaluation studies. As there were more studies in the literature, a choice had to be made as to which studies to include in this review. Two important inclusion criteria were used: first, the included meta-analytic studies should be systematic, i.e., use systematic methods to collect secondary data and critically appraise these studies [109]; second, the included studies should be RCTs, as only a randomized control group design allows for drawing valid conclusions about the effectiveness of Qigong. Because the findings of included and excluded studies are similar, the studies mentioned in this overview can be considered to represent a larger set of research.

A second limitation relates to the quality of Qigong research. As this is a narrative review, no reference is made to the quality and effect sizes in the studies. Although most studies in this review are RCTs, they often make use of relatively small groups with a limited range of participants, such as women, patients, or older participants, which may limit generalizability. To avoid this issue, the present review has tried to also refer to studies with healthy, younger and/or working individuals (e.g., [63,71,73,74,82]). While the outcomes of these studies are similar, more research is needed that includes a broader range of practitioners.

Third, most study findings were positive, presenting Qigong as an effective intervention. Only in a few cases, a null-finding was reported. For instance, Hwang et al. [72] observed how a four-week Qigong training program resulted in less perceived stress but not in reduced cortisol levels. Jahnke et al. [18] found that some studies did not detect significant differences in depression and anxiety in response to Qigong. These predominantly positive results may be the result of a publication bias. To obtain a more comprehensive view of the effects of Qigong, future meta-analyses might try to also cover unpublished research findings.

Several additional directions for future research deserve mentioning. As previous research has primarily focused on the curative effects of Qigong, future research could investigate Qigongs’ preventive effects. Because practicing Qigong contributes to a state of mental relaxation and improved cognitive functioning, Qigong practitioners are more likely to appraise stressful situations in a positive way [83], are less likely to develop stress sensitization [10], and generally might develop a more relaxed attitude to life [19]. As such, practicing Qigong may unset an upward spiral process. More research is needed to investigate these propositions and determine which mechanisms, such as a decreased activation level, enhanced cognitive processing, or improved affect regulation, explain these effects.

New research could also investigate the consequences of Qigong practice other than health outcomes, such as interpersonal relationships. If Qigong practitioners develop a more relaxed stance in life and improved affect regulation, this might affect the quality of the relationships they have with important others, such as family, friends, colleagues, subordinates, and clients. In turn, these relationships can have important outcomes. In mindfulness research, for instance, associations have been found between leaders’ mindfulness with subordinates’ job satisfaction, need satisfaction, and job performance [110]. Qigong research could similarly focus on such indirect outcomes of Qigong practice at work and outside work.

Another important issue relates to individuals’ participation in Qigong training. Health effects can only be achieved with regular Qigong training that continues over time [20]. Very few studies have focused on individuals’ motivation to start Qigong training or the factors that predict whether they will adhere to the training or drop off. As an exception, Jouper [111] found that adherence was greater when participants were highly intrinsically motivated and experienced more wellness during the training. More research is needed to better understand the behavior of practitioners with regard to starting and persisting with Qigong, and to provide more information about the individuals for whom Qigong is an attractive training method (or not).

### 4.3. Practical Implications: Qigong’s Role in Organizational Stress Management Programs

This review has also implications for practice, and in particular for stress management in organizations. Despite the adverse effects of stress on individuals, employers pay relatively little attention to understanding the sources of work-related stress and alleviating stress symptoms compared to other areas, such as technological developments and increased international competition [112]. Measures to reduce stress at work are often taken after negative organizational outcomes, such as high absenteeism or staff turnover, have occurred [113]. To deal with occupational stress, different stress management approaches have been developed. The most common approach is to provide curative interventions aimed at easing the strain of troubled employees, which can be considered a reactive approach, as the health problem already exists. In contrast, preventive approaches aim to forestall the development of strain by either focusing on the stressful work situation and trying to reduce the sources of stress (i.e., stressors), or focusing on employees, for instance by teaching them techniques to reduce the psychological and physiological symptoms of stress before they become troublesome [114].

Qigong can contribute to both curative and preventive stress management programs. Quite some studies in this review have shown positive effects of practicing Qigong on participants who already suffered from stress-related symptoms such as hypertension, sleep disorders, anxiety, and depression (e.g., [20]). In such a curative approach, practicing Qigong can help reduce these symptoms and support participants in regaining their health. At the same time, Qigong can make an important contribution to preventive stress management programs by decreasing daily sympathetic activity, stimulating parasympathetic activity, and increasing individuals’ positive life experiences. This preventive role is in line with traditional Chinese Medicine, which aims to keep all aspects of a person in balance so that disease has no chance to develop. Recognizing the importance of a healthy workforce, some organizations have already established workplace wellness programs that focus on, for instance, lifestyle improvement. Qigong could be a valuable addition to these programs.

Organizations can contribute to Qigong practice at different levels. At the workplace level, supervisors and coworkers can support each other by accepting, encouraging, and facilitating Qigong practices. Leaders or middle managers who are responsible for workers’ well-being could provide workers with the autonomy and opportunity to incorporate Qigong into their daily schedule. At the organizational level, top management could establish a healthy work culture that emphasizes the role of wellness programs in general and Qigong practice in particular. At the same time, and in line with traditional Chinese Medicine’s emphasis on individuals’ responsibility for their own health [45], workers will have to show initiative and perseverance, reserve some time for Qigong practice, and have the discipline, or courage, to put that important task or their mobile phone away for a while. As this review of Qigong research indicates, health effects are already achieved with a small number of short Qigong sessions per week, provided they are continued over time. A supportive social environment can be helpful for Qigong adherence [115]. As the World Health Organization emphasizes, a healthy workplace can only be realized if workers and managers collaborate [116].

## 5. Conclusions

Studies with RCTs have shown that practicing Qigong impacts the effects of stress and overactivation by decreasing stress levels, hypertension, depression, and anxiety, and improving the cardiovascular and respiratory systems, immune function, sleep quality, cognitive functioning, and stress appraisal. Four elements of Qigong appear to contribute to these positive effects, i.e., physical training, active relaxation, focused attention, and conscious breathing. Together, extensive empirical evidence suggests that Qigong provides an effective means for individual stress prevention that individuals can use to recover from stress and sustained activation, regain homeostasis and mind–body balance, and improve their physical and psychological well-being.

## Figures and Tables

**Table 1:** Summary of Stress-Reducing Outcomes of Qigong and its Components ^1^.

Outcomes	Source ^2^
Physical Health Outcomes	
Reduced Hypertension	[18,51,57,61,62,64,88]
Improved Cardiovascular System	[18,46,66,87,93]
Improved Respiratory System	[46,51,63]
Improved Immune System	[18,67,68,69,96,97,98]
Reduced Levels of Stress Hormones	[42,65,70,74,86,93,102]
Mental Health Outcomes	
Reduced Stress Perceptions	[70,71,72,73,91,93,96,97,98]
Improved Sleep Quality	[18,20,21,63,93,106]
Reduced Fatique	[75,76,94,96,97,98]
Reduced Anxiety	[56,60,63,70,73,77,92,93,96,97,98,102]
Reduced Depression	[20,42,60,63,78,79,80,81,96,97,98,102]
Improved Cognitive Functioning	[47,82,89,101,104,107]
Improved Appraisal	[13,83,84]
Decreased Stress-Sensitization	[13,89]

Notes: ^1^ These effects are achieved with 30–60 minute training, 2–3 times per week, during 8–16 weeks. ^2^ Sources pertain to the references in this review.
